# Period of Measurement in Time-Series Predictions of Disease Counts from 2007 to 2017 in Northern Nevada: Analytics Experiment

**DOI:** 10.2196/11357

**Published:** 2019-01-15

**Authors:** Amir Talaei-Khoei, James M Wilson, Seyed-Farzan Kazemi

**Affiliations:** 1 Department of Information Systems University of Nevada Reno Reno, NV United States; 2 School of Software University of Technology Sydney Sydney Australia; 3 Nevada Medical Intelligence Center School of Community Health Sciences and Department of Pediatrics University of Nevada Reno Reno, NV United States; 4 Center for Research and Education in Advanced Transportation Engineering Systems Rowan University Glassboro, NJ United States

**Keywords:** autocorrelation, disease counts, prediction, public health surveillance, time-series analysis

## Abstract

**Background:**

The literature in statistics presents methods by which autocorrelation can identify the best period of measurement to improve the performance of a time-series prediction. The period of measurement plays an important role in improving the performance of disease-count predictions. However, from the operational perspective in public health surveillance, there is a limitation to the length of the measurement period that can offer meaningful and valuable predictions.

**Objective:**

This study aimed to establish a method that identifies the shortest period of measurement without significantly decreasing the prediction performance for time-series analysis of disease counts.

**Methods:**

The data used in this evaluation include disease counts from 2007 to 2017 in northern Nevada. The disease counts for chlamydia, salmonella, respiratory syncytial virus, gonorrhea, viral meningitis, and influenza A were predicted.

**Results:**

Our results showed that autocorrelation could not guarantee the best performance for prediction of disease counts. However, the proposed method with the change-point analysis suggests a period of measurement that is operationally acceptable and performance that is not significantly different from the best prediction.

**Conclusions:**

The use of change-point analysis with autocorrelation provides the best and most practical period of measurement.

## Introduction

### Overview

In a time-series prediction for a population, a measurement is the record of equally spaced disease counts over time. The length of these measurements, or equivalently, the interval between records, is the period of measurement [1]. Although time-series predictions have been widely used in public health surveillance, a body of literature in statistics presents methods by which autocorrelation can detect the best periodicity. Periodicity detection refers to the detection of periodic patterns in a time-series database [2] and can improve the performance of time-series prediction [3-5]. Autocorrelation is a measure of the internal correlation within a time series [4] and a way of measuring and explaining internal association between observations in a time series. The autocorrelation sequence of a periodic time series has the same cyclic characteristics as the time series itself. Thus, autocorrelation can help verify the presence of cycles and determine their durations [3]. Autocorrelation is often used to identify the best periodicity for time-series analysis [3-5]. This method selects the periodicity in which the autocorrelation is maximized, which would provide time series with better prediction performance [5]. The main aim of this study was to establish a period of measurement in which the autocorrelation is maximized and the periodicity is the interval that its prediction outperforms.

The selection of the period of measurement determines the interval for prediction. Thus, from the operational perspective, for the prediction to be meaningful and valuable in public health surveillance, there has to be a limit to its length. For example, when predicting influenza A cases for the next year, although the 8-week period of measurement may generate the best performance for the prediction, it also produces predicted values that are aggregated 8 weeks at a time. This period is too long to provide any value for practitioners. The 8-week period would cover most of the winter, which is expected to have more influenza A cases even if there is no prediction. For many diseases, particularly infectious diseases that are not disruptive to the healthcare infrastructure (eg, influenza), a 1-week prediction window is generally sufficient. However, for the healthcare infrastructure, a greater prediction window would be helpful to allow planning for potential changes, staffing, or resource allocation. Ultimately, identification of the optimal prediction window allows users to decide what is acceptable for their role in the community.

In response to the operational concern discussed above, this study aims to identify the shortest period of measurement without significantly decreasing the performance. Although autocorrelation provides the best period of measurement, this period may be too long to be practically acceptable. Therefore, we adopted a change-point analysis (CPA) to detect a shorter period of prediction with no change point in between in order to achieve similar performance with a shorter period. To this end, our method aims to apply CPA for autocorrelations of different periods of measurement. The objective is to identify the shortest period of measurement that has an autocorrelation value similar to the maximum value of autocorrelations.

### Background and Significance

#### Public Health Surveillance

The initial target of public health surveillance was infectious diseases; however, with the recent advancements in analytics, data from surveillance systems are increasingly used to predict future trends in a wide range of noninfectious disease distributions. Data have been used for further resource planning and initiating warning systems [6,7]; for example, the Centers for Disease Control and Prevention organized a challenge to predict the 2013-2014 United States influenza season [8]. The ability to accurately forecast various diseases could facilitate key preparedness actions such as the development and use of medical countermeasures, communication strategies, and healthcare resource management [9]. To achieve this goal, different statistical methods have been used to forecast disease counts; time-series prediction is a method often used in relevant literature [1,10-12], wherein the analysis predicts disease counts by modelling historical surveillance data [1,13]. However, the literature in this area recommends the use of a wide range of methods such as Autoregressive Integrated Moving Average (ARIMA) [14] and structural equation modelling [15].

#### Time-Series Prediction in Public Health Surveillance

Prior work in time-series prediction of public health surveillance has heavily relied on aberrancy-detection algorithms that are used to detect temporal changes in the data, which may be indicative of a disease outbreak [16]. The Centers for Disease Control and Prevention's Early Aberration Reporting Systems uses C algorithms. In terms of prediction capabilities, C1 only supports moving average with a 7-day window, whereas C2 and C3 offer moving average with a 7-day window and 2-day guard band. Similar to C1-C3, other algorithms [17-19] do not have long-term predictive features that allow public health authorities to achieve annual planning.

These algorithms are primarily designed on the basis of conventional hypothesis testing for the existence of disease outbreak. Aberrancy-detection algorithms only detect changes in static disease activity at a given time when the outbreak occurs and only notice the direction of changes in disease trends at a single time point [20]. However, when the prediction is for an annual disease count rather than a disease outbreak, ARIMA models and machine learning can address the limitation of aberrancy-detection algorithms [21].

ARIMA models are commonly used in public health surveillance [14] and are built on three basic ideas: (1) the present value of time-series is a linear function of its past values and random noise in the AR model [22], (2) the present value of time-series is a linear function of its present and past values of residuals in the moving average model [23], and (3) the AR moving average model [24] considers both the AR and moving average models as well as the historical values and residuals. The ARIMA model generally fits the time-series data based on a previous AR moving average model [24] and includes a differentiating process that effectively transforms nonstationary data required for the abovementioned models into stationary data used in ARIMA [14]. The ARIMA models have been widely used for time-series prediction in public health surveillance [13,25], including hemorrhagic fever with renal syndrome [26,27], dengue fever [28], tuberculosis [29], and mental health [30].

Although methods in conventional statistics are designed to assign most importance to immediate data, they work better with short-term predictions. In addition, these techniques are based on the notion that relationships among the constructs would continue in future, which may not be true [30]. A growing body of literature [31-35] addressed this issue through the use of machine-learning approaches such as Artificial Neural Networks (ANNs) for time-series prediction in public health surveillance. ANNs are inspired by the ways in which biological nervous systems such as the brain process information. It is composed of a large number of highly interconnected processing elements (similar to neurons) working in unison to recognize patterns in data. In addition, ANNs, like people, learn by example.

The ability of ANN to recognize patterns in data allows for better predictions and provides assistance for public health surveillance because it is able to self-organize and self-learn processes [36]. Public health surveillance uses ANN to forecast diseases distributions, whereas Guan et al (2004) used ANN to forecast incidents of hepatitis. Mehra et al (2016) also used ANN to predict the preplanting risk of *Stagonospora nodorum* blotch in winter wheat.

Since this study focuses on forecasting disease counts and limitations of aberrancy-detection algorithms to detect disease outbreak, we only discuss ARIMA and machine learning here.

### Period of Measurement for Time-Series Prediction in Public Health Surveillance

Several studies have focused on predicting diseases for public health surveillance through the use of time-series methods such as ARIMA and machine learning. However, it is necessary to recognize that measurement periods play a significant role in the performance of time series, as time-series prediction methods may show different performances for the same population when predicting in different measurement periods [37-39]. For better surveillance of a disease, it is crucial to identify the period of measurement in which the time-series methods demonstrate the best performance for prediction in a particular population.

The performance indicators for time series, such as Q-score [40], can be used to identify the period of measurement that generates the best performance. However, they are computationally expensive to run across multiple time-series analysis for different periods of measurement and compare the performance using the indicator. Therefore, the literature in this field has suggested autocorrelation as one of the most commonly used algorithms to identify the best period of measurement in time series [5]. Autocorrelation refers to the correlation of a time series with its own past and future values [3]. The main objective of this method is to obtain an autocorrelation sequence of a periodic signal with the same cyclic characteristics as the signal itself, allowing autocorrelation to verify the presence of cycles and determine their durations [4]. Therefore, the overall goal is to determine the period of measurement that maximizes the autocorrelation to provide better performance prediction [5].

Although autocorrelation may suggest a periodicity mapped to a period of measurement that is operationally too long to be meaningful, the current study aims to use CPA in order to identify the shortest period of measurement with an autocorrelation value similar to the maximum autocorrelation value. Therefore, we do not expect to see a significant drop in the performance prediction.

## Methods

### Change-Point Analysis

CPA is exclusively designed to detect subtle changes and characterize changing trends in a time-series [20,41]. The literature has proposed several methods of CPA such as standard normal homogeneity, two-phase regressions with a common trend, and penalized likelihood criteria. In this study, we used the pruned exact linear time (PELT) CPA method suggested by Killick et al (2012) [42]. This method is based on the CPA method of Jackson et al (2005) [43], but incorporates a pruning step that reduces the computational cost of the method and does not affect the exactness of the resulting segmentation. Although many CPA methods that can only detect the most significant change point, PELT can identify multiple change points. Therefore, owing to its computational performance, this study adopted the PELT method [44]. In addition, we used the R package for CPA [45], which implements PELT. In this algorithm, a change point is defined as the point that characterizes changing trends. As such, the value for the change point is significantly different from the point value immediately before the change point.

The PELT algorithm uses a common approach to detect change points through minimization of costs, which improves the computation performance of PELT. To find multiple change points, the PELT algorithm is first applied to the whole dataset and iteratively and independently to each partition until no further change points are detected. The main assumption of the PELT algorithm is that the numbers of change points increase linearly with the increase in the dataset; the change points are spread throughout the data and are not restricted to one portion of the data [44]. Since we used a small dataset in this study, this assumption is met.

### Proposed Method

Our method sorts the autocorrelations based on their period of measurements, wherein the autocorrelation for the shortest period of measurement occupies the first place and the autocorrelation for the longest period of measurement occupies the last place. After conducting CPA using the PELT algorithm on autocorrelations, our method indicates the immediate ascending change point (ACP) before the highest autocorrelations. The autocorrelation of the ACP is the autocorrelation for the shortest period of measurement with similar performance as the highest autocorrelation. Since ACP indicates the closest ACP to the highest autocorrelations, there will be no ACP between the ACP and the highest autocorrelations. This would result in similar performance between the period of measurement associated with the ACP and the period of measurement for the highest autocorrelations. In addition, this will be the shortest period of measurement with similar performance as the highest autocorrelations, because we skip all periods of measurements between the ACP and the highest autocorrelations. As such, the ACP is the shortest period of measurement that has similar performance as the highest autocorrelations.

**Figure 1:**
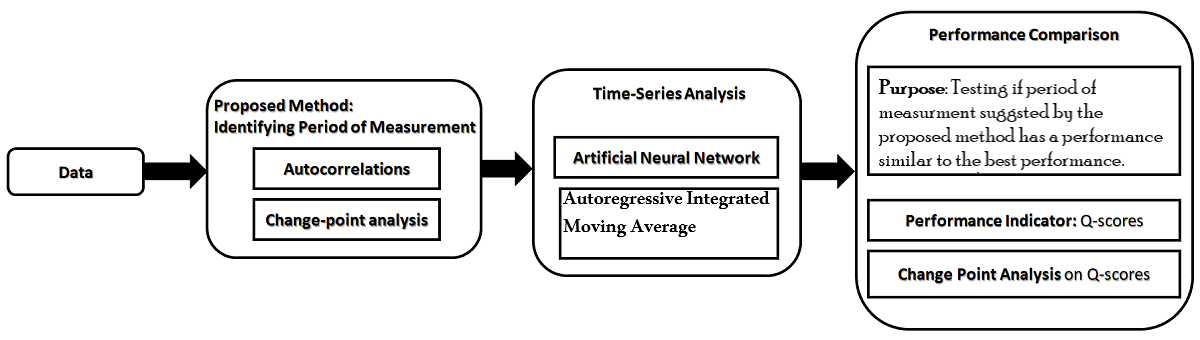
Evaluation of the proposed method.

If the immediate change point before the highest autocorrelations is descending, there is no available period of measurement that is shorter than the highest autocorrelations and has similar performance as the highest autocorrelation. Therefore, the highest autocorrelations indicates the aimed period of measurement. If there is no change point before the highest autocorrelation, we consider the first point as the immediate change point prior to the highest autocorrelation. Figure 1 presents the evaluation of the proposed method.

### Data Description

We used the notifiable disease case counts by epidemiological week from 2007 to 2017 in Washoe, Clark, and Carson Counties in Northern Nevada. The data included case counts for chlamydia, salmonella, respiratory syncytial virus (RSV), gonorrhea, viral meningitis, and influenza A. The data were deidentified and included patients of all age. For each disease, the dataset provides the number of reported cases in each epidemiological week. Therefore, for each week between 2007 and 2017, the dataset included all the reported cases of the abovementioned diseases in the three counties, separated according to the diseases.

### Training and Test Datasets

The data were divided into training and test datasets in the ratio of 10:1. The scaling guidelines proposed by Guyon [46] were adopted to identify the size of the training and test sets. The time-series analysis was trained using the dataset created from the data of 2007-2016 and tested on the data for 2017. The performance was subsequently reported.

**Figure 2:**
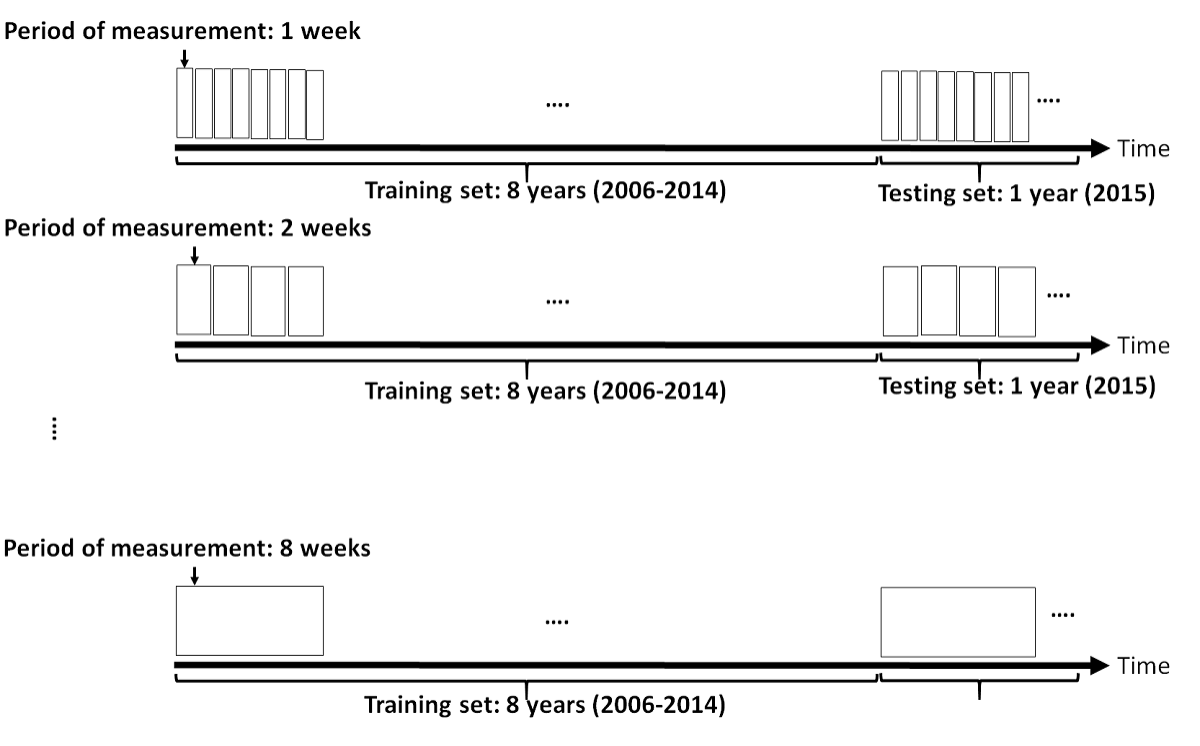
Training and testing sets.

The original datasets, mentioned in the Data Description section, are measured at 1-week periods. Therefore, the minimum period of measurement was 1 week. However, the study evaluated periods of measurement from 1 to 8 weeks. Depending on the period of measurement, the training and testing sets were aggregated into groups of 1-8 weeks. For example, when we look at the 3-week measurements, the 1-week measurements are aggregated into groups of three. This aggregation starts from week 1. Figure 2 presents the training and testing sets for period measurements.

### Time-Series Analysis

**Figure 3:**
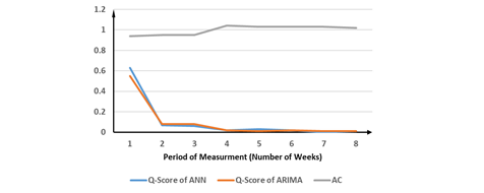
Evaluation of the proposed method for chlamydia cases. ANN: Artificial Neural Network; ARIMA: Autoregressive Integrated Moving Average; AC: ascending change.

**Figure 4:**
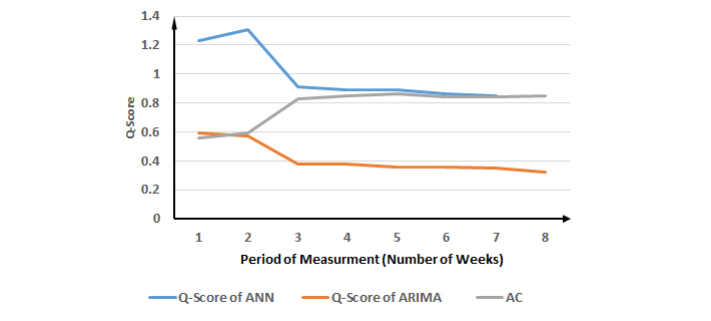
Evaluation of the proposed method for salmonella cases. ANN: Artificial Neural Network; ARIMA: Autoregressive Integrated Moving Average; AC: ascending change.

**Figure 5:**
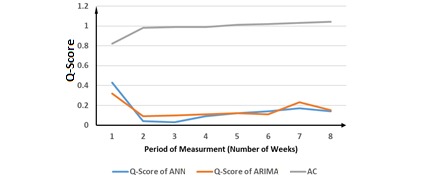
Evaluation of the proposed method for respiratory syncytial virus cases. ANN: Artificial Neural Network; ARIMA: Autoregressive Integrated Moving Average; AC: ascending change.

**Figure 6:**
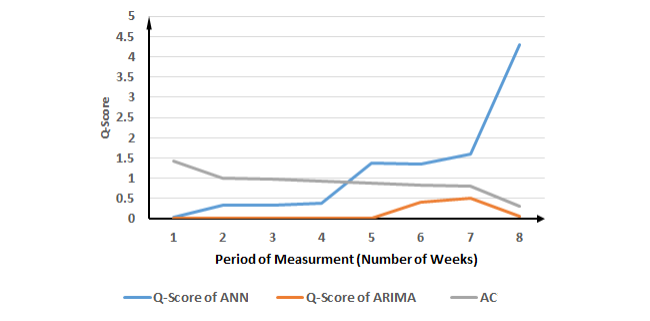
Evaluation of the proposed method for gonorrhea cases. ANN: Artificial Neural Network; ARIMA: Autoregressive Integrated Moving Average; AC: ascending change.

**Figure 7:**
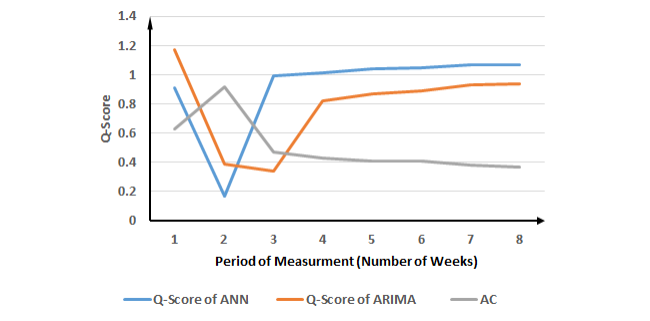
Evaluation of the proposed method for viral meningitis cases. ANN: Artificial Neural Network; ARIMA: Autoregressive Integrated Moving Average; AC: ascending change.

**Figure 8:**
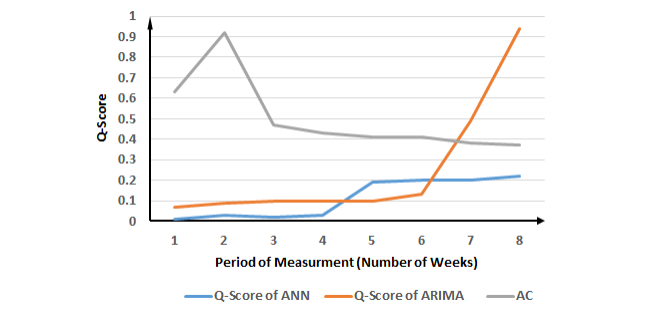
Evaluation of the proposed method for influenza A cases. ANN: Artificial Neural Network; ARIMA: Autoregressive Integrated Moving Average; AC: ascending change.

In order to implement ARIMA, we used auto.arima from the R package of forecast [47]. Considering the growing body of literature on ANN for public health surveillance [36,48,49], we selected the ANN model for machine learning. Depending on the learning structure, there are many different types of ANNs. In this study, we adopted a feed-forward perceptron-based ANN [50] implemented by the R package CRAN: nnet (version, 7.3-5), as it was the most-suitable ANN for our data structure in the preliminary analysis. The parameters were model=multinomial log-linear models: maximum number of iterations=100, fitting=least squares, initial random weights=0.7, maximum allowable number of weights=1000, absolute stop fit criterion=1.0^e-4^, relative stop fit criterion=1.0^e-8^, size of single hidden layers=11, and weight decay=0.1. These parameters were run for each disease separately, and the predictor variable was time measured by the period of measurement. Figures 3-8 present the performance of ANN and ARIMA.

### Performance Indicator: Q-Score

**Figure 9:**
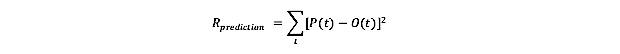
Prediction error.

The performance of time-series analysis was measured using the Q-score indicator proposed by Ghil et al (2011) [40]. This indicator treats the data as continuous data, and therefore, the predicted value or observed value can be any positive number in the testing set. Formally, for each disease under the evaluation, we consider the prediction values of P(*t*)∈[0,∞) and the observation values of 0(*t*)∈[0,∞) with integer time 1≤t≤52 counting weeks within a year. The overall error of the prediction is quantified by the total squared discrepancy between the prediction values and observed values for the testing set (Figure 9).

**Figure 10:**
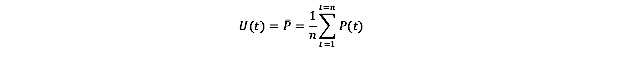
Historic average.

To evaluate the performance of prediction, we compared the time-series analysis under evaluation with the unskilled prediction that predicts constant historic average count. This formula is defined in Figure 10.

**Figure 11:**
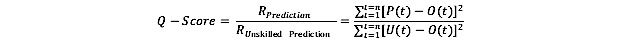
Q-Score.

Finally, the Q-score was defined as the quadratic errors of prediction under evaluation and the unskilled prediction presenting as a constant average. Therefore, the Q-score was defined as presented in Figure 11.

The Q-score may take positive values. It takes Q–score=1 if the time-series prediction under evaluation generates similar results as the unskilled prediction, producing a constant average. A desired time-series analysis produces Q–score=1. Therefore, the aim was to minimize the Q–score.

The Q-score for each period of measurement was calculated for both ARIMA and ANN. Subsequently, a CPA was conducted to determine if the suggested period of measurement generated similar performance as the best performance prediction generating the smallest Q-score with ARIMA and ANN.

This provides a comparative indicator to show the extent to which a method improves unskilled random prediction, which fits our study requirements. The Q-score uses unskilled prediction as a basis and demonstrates how a method outperforms an unskilled prediction. Therefore, the Q-score is suitable for our purpose of comparing methods.

## Results

Figure 3 depicts the evaluation of the proposed method for chlamydia cases. The results show that the proposed method suggests a period of measurement of <3 weeks, which is operationally acceptable. Our result was validated against the performance of ANN and ARIMA, measured by the Q-score (Table 1).

Figure 3 and Table 1 present the evaluation of the proposed method for chlamydia cases. The biggest AC is for the 4-week period of measurement. However, the immediate ACP is in the 2-week period of measurement. Therefore, the autocorrelations are similar in the 2- to 4-week periods of measurements. The proposed method suggests that the 2-week period of measurement yields a good performance, similar to the best performance. The best performance measured by Q-score occurs in 7-week period of measurement for ANN and the 5-week period of measurement for ARIMA. Although there is no ACP, the descending change point (DCP) is in the 2-week period of measurement. As such, performance of ANN and ARIMA remained similar for the 2-week period of measurement or longer. Although the 7-week period of measurement for ANN and 5-week period of measurement for ARIMA provided the best performance, and the smallest Q-scores, our results show that the 2-week period of measurement indicated by our proposed method showed similar performance.

Although our proposed method suggests the 3-week period of measurement for salmonella cases, the best performance occurs in the 8-week period of measurement for both ANN and ARIMA (Figure 4 and Table 2). However, the results of CPs on Q-scores shows that that 3-week period of measurement generates similar performance as the best Q-scores for ANN and ARIMA. The results for RSV (Figure 5 and Table 3) and gonorrhea cases (Figure 6 and Table 4) validate the proposed method.

Figure 7 and Table 5 demonstrate an interesting example for viral meningitis. The 2-week period of measurement was suggested by the proposed method, whereas the highest and ACP for AC occurs in the 2-week period of measurement. For ANN, the best performance measured by the Q-score occurs for the 3-week period of measurement; however, the 2-week period of measurement shows a DCP for the Q-scores of ANN. Therefore, the 3-week period of measurement generates similar performance as the 2-week period of measurement suggested by the proposed method. For ARIMA, the best performance occurs in the 2-week period of measurement, which has the DCP as well. The proposed method was also validated for viral meningitis.

Influenza A has attracted a lot of attention from time-series analysis in public health. The biggest AC occurs in the 2-week period of measurement, but the best performance is in the 1-week period of measurement for both ANN and ARIMA. However, there is no change point until the 5-week period of measurement for ANN and the 7-week period of measurement for ARIMA when ACP occurs. Therefore, we can assume that in both ANN and ARIMA, the performance of the 1-week period of measurement with the best Q-score is similar to that of the 2-week period of measurement suggested by the proposed method, because of the biggest AC with the DCP in the 2-week period of measurement (Figure 8 and Table 6). In addition, the proposed method improves the prediction of influenza A.

**Table 1:** Validation of the proposed method for chlamydia cases against the performance of Artificial Neural Networks and Autoregressive Integrated Moving Average, measured by the Q-score.

Period of measurement (week)	Q-score of the Artificial Neural Networks	Q-score of the Autoregressive Integrated Moving Average	Ascending change
1	0.63	0.55	0.94
*2*	0.07 (DCP^a^)	0.08 (DCP^a^)	0.95 (ACP^b^)
3	0.06	0.08	0.95
4	0.02	0.02	1.04^c^
5	0.03	0.01^d^	1.03
6	0.02	0.02	1.03
7	0^d^	0.01	1.03
8	0.01	0.01	1.02

^a^DCP: descending change point.

^b^ACP: ascending change point.

^c^Biggest ascending change.

^d^The best performance measured by the Q-score for Artificial Neural Networks and Autoregressive Integrated Moving Average.

**Table 2:** Validation of the proposed method for salmonella cases against the performance of Artificial Neural Networks and Autoregressive Integrated Moving Average, measured by the Q-score.

Period of measurement (week)	Q-score of the Artificial Neural Networks	Q-score of the Autoregressive Integrated Moving Average	Ascending change
1	1.23	0.59	0.56
2	1.31	0.57	0.59
*3*	0.91 (DCP^a^)	0.38 (DCP^a^)	0.83 (ACP^b^)
4	0.89	0.38	0.85
5	0.89	0.36	0.86^c^
6	0.86	0.36	0.84
7	0.85	0.35	0.84
8	0.82^d^	0.32^d^	0.85

^a^DCP: descending change point.

^b^ACP: ascending change point.

^c^Biggest ascending change.

^d^The best performance measured by the Q-score for Artificial Neural Networks and Autoregressive Integrated Moving Average.

**Table 3:** Validation of the proposed method for respiratory syncytial virus cases against the performance of Artificial Neural Networks and Autoregressive Integrated Moving Average, measured by the Q-score.

Period of measurement (week)	Q-score of the Artificial Neural Networks	Q-score of the Autoregressive Integrated Moving Average	Ascending change
1	0.43	0.32	0.82^a^
*2*	0.04 (DCP^b^)	0.09^c^ (DCP^b^)	0.98 (ACP^c^)
3	0.03^d^	0.1	0.99
4	0.09	0.11	0.99
5	0.12	0.12	1.01
6	0.14	0.11	1.02
7	0.17	0.23	1.03
8	0.14	0.15	1.04

^a^Biggest ascending change.

^b^DCP: descending change point.

^c^ACP: ascending change point.

^d^The best performance measured by the Q-score for Artificial Neural Networks and Autoregressive Integrated Moving Average.

**Table 4:** Validation of the proposed method for gonorrhea cases against the performance of Artificial Neural Networks and Autoregressive Integrated Moving Average, measured by the Q-score.

Period of measurement (week)	Q-score of the Artificial Neural Networks	Q-score of the Autoregressive Integrated Moving Average	Ascending change
1	0.04^a^	0.01^a^	1.42^b^
2	0.33	0.02	1.01
3	0.34	0.02	0.98
4	0.39	0.02	0.93
5	1.38	0.02	0.88
6	1.36	0.4	0.82
7	1.59	0.5	0.81
8	4.3	0.05	0.31

^a^The best performance measured by the Q-score for Artificial Neural Networks and Autoregressive Integrated Moving Average.

^b^Biggest ascending change.

**Table 5:** Validation of the proposed method for viral meningitis cases against the performance of Artificial Neural Networks and Autoregressive Integrated Moving Average, measured by the Q-score.

Period of measurement (week)	Q-score of the Artificial Neural Networks	Q-Score of the Autoregressive Integrated Moving Average	Ascending change
1	0.91	1.17	0.63
*2*	0.17^a^ (DCP^b^)	0.39 (DCP^b^)	0.92^c^ (ACP^d^)
3	0.99	0.34^a^	0.47
4	1.01	0.82	0.43
5	1.04	0.87	0.41
6	1.05	0.89	0.41
7	1.07	0.93	0.38
8	1.07	0.94	0.37

^a^The best performance measured by the Q-score for Artificial Neural Networks and Autoregressive Integrated Moving Average.

^b^DCP: descending change point.

^c^Biggest ascending change.

^d^ACP: ascending change point.

**Table 6:** Validation of the proposed method for influenza A cases against the performance of Artificial Neural Networks and Autoregressive Integrated Moving Average, measured by the Q-score.

Period of measurement (week)	Q-score of the Artificial Neural Networks	Q-score of the Autoregressive Integrated Moving Average	Ascending change
1	0.01^a^	0.07^a^	0.63
*2*	0.03	0.09	0.92^b^ (ACP^c^)
3	0.02	0.10	0.47
4	0.03	0.1	0.43
5	0.19 (ACP^c^)	0.1	0.41
6	0.2	0.13	0.41
7	0.2	0.49 (ACP^c^)	0.38
8	0.22	0.94	0.37

^a^The best performance measured by the Q-score for Artificial Neural Networks and Autoregressive Integrated Moving Average.

^b^Biggest ascending change.

^c^ACP: ascending change point.

## Discussion

Following the extensive use of time-series predictions in public health surveillance, autocorrelation is commonly used in statistics to identify the best period of measurement and improve the performance of predictions [3-5]. However, the forecast needs to address the operational perspective and offer meaningful and valuable predictions. Therefore, practitioners in public health surveillance may choose a shorter period of measurement wherein the forecast results may not be as accurate as those of analyses of longer periods of measurements. The literature in statistics shows how the best period of measurement suggested by autocorrelation can improve the performance of a time-series prediction [3-5]. In addition, our empirical results revealed that the most-outperforming period of measurement is not always the shortest one. However, the long periods of measurement that likely provide better prediction performance may not be useful to practitioners because they are too long. We have provided examples of such instances in the Introduction section of the manuscript.

This study proposed a method that runs CPA on autocorrelations and identifies the shortest period of measurement with a performance prediction similar to the best performance prediction. Our method was evaluated against ANN and ARIMA methods for a time-series analysis of disease counts in Cark, Carson, and Washoe Counties in Northern Nevada between 2007 and 2017, including case counts for chlamydia, salmonella, RSV, gonorrhea, viral meningitis, and influenza A.

Unfortunately, autocorrelation cannot guarantee the best performance for disease prediction. For example, for chlamydia, the greatest autocorrelation occurred in the 4-week period of measurement, the best performance of ANN was noted in the 7-week period, and the best performance of ARIMA was observed in the 5-week period of measurement. This was also the case for RSV, gonorrhea, viral meningitis, and influenza A. However, the proposed method adopting CPA suggests that the shortest period of measurement (to satisfy operational perspective) ensures acceptable performance predictions similar to the best Q-scores.

The current study has two implications for academics. First, the study adds information on the importance of the period of measurement as a factor for providing better disease count forecasts. Second, it demonstrates the application of CPA in providing operationally focused autocorrelation for a more practical period of measurement that not only improves the prediction performance but also generates practical insights.

From a practical perspective, time-series prediction is an important tool for public health and clinical medicine to identify seasonal periods of changes in the relative risk for disease activity. Observed values that exceed predicted parameters do not necessarily reflect a “failed” prediction, but rather, a pattern of reported activity that was not observed in previous data. This is an important adjunct to other methodologies for aberration detection, such as the aforementioned Early Aberration Reporting System. Predictions offer value to the unaware practitioner by offering a “most likely” hypothesis for expected disease activity, which may carry implications for proactive education and disease-control policies.

Although the current study evaluated the proposed method for a variety of diseases, the data were limited to Northern Nevada. Therefore, expanding the datasets and re-evaluating the method with a wider range of diseases from various geographical locations and larger sample sizes would provide a better understanding of the performance prediction of this method. In addition, the proposed method was evaluated against only ARIMA and ANN. This limitation can be addressed in future studies by applying more time-series prediction methods. Although this method uses autocorrelation, Fourier Transforms have been used in the literature to identify the period of measurement [51]. Thus, further research can compare the performance of AC and Fourier Transforms adopted in the method proposed in this study. In addition, the use ARIMA as a predictive model despite its difficulties with periodic prediction has limited the evaluation of our study. However, the purpose of this study was to compare ANN and ARIMA for their applicability with the method proposed.

Although we chose ARIMA and ANN to demonstrate the performance of the suggested method, researchers in this field are encouraged to use other conventional or machine-learning algorithms to evaluate the performance of this method in future.

The study has potential from a mathematical perspective, since the different time series generated by autocorrelation are mathematical manipulations of the original time series. For example, they could be modelled as reindexed discrete-time stochastic processes. This would open an avenue of future research to mathematically study the behavior of these time series.

The period of measurement plays an important role in the performance of time-series analysis for disease counts. The literature in statistics has been using autocorrelation to identify the outperforming period of measurement. However, in predicting disease counts, long periods may not provide sufficient values for public health and surveillance practitioners. Therefore, we used CPA to find the shortest period of measurement, which has similar performance as the period identified by AC.

In conclusion, through the adoption of autocorrelation and CPA, we propose a novel method for identifying the period of measurement, which can improve the performance of time-series predictions for disease counts. Our method implements a practical perspective through which we aim to determine the shortest period of measurement that achieves a better prediction performance. This finding makes the method practically applicable in the field when longer periods of prediction, even with better performance, are not operationally valuable to public health professionals. Our method was evaluated against ANN and ARIMA analyses for disease counts of chlamydia, salmonella, RSV, gonorrhea, viral meningitis, and influenza A between 2007 and 2017 in Northern Nevada. Future work should focus on enhancing the evaluation of the method by using more diverse datasets as well as assessing the use of Fourier Transforms instead of AC. Moreover, we encourage researchers to use a wide range of machine learning and alternative CPA methods to improve the suggested approach.

## Abbreviations

ACP: ascending change point
ANN: Artificial Neural Network
ARIMA: Autoregressive Integrated Moving Average
CPA: change-point analysis
DCP: descending change point
PELT: pruned exact linear time
RSV: respiratory syncytial virus
